# Boundary optimization of inclined coal seam open-pit mine based on the ISSA–LSSVR coal price prediction method

**DOI:** 10.1038/s41598-023-34641-7

**Published:** 2023-05-09

**Authors:** Bo Cao, Shuai Wang, Runcai Bai, Bo Zhao, Qingyi Li, Mingjia Lv, Guangwei Liu

**Affiliations:** College of Mining, Liaoning Technical University, Fuxin, 123000 China

**Keywords:** Engineering, Mathematics and computing

## Abstract

As an important link in the complex system engineering project of open pit mining, the quality of the boundary determines the performance of the project to a large extent. However, changes in economic indicators may raise doubts about the optimality of mining boundaries. In this article, a coal price time series forecasting model that considers some amount of redundancy is proposed, which combines an improved sparrow search algorithm (ISSA) and a least squares support vector regression machine regression (LSSVR) algorithm. The optimal values of the penalty factor and kernel function parameter of the LSSVR model are selected by ISSA, which improves the prediction accuracy and generalization performance of the forecasting model. A multistep decision optimization method under fluctuating coal price conditions is proposed, and the model prediction results are applied to the boundary optimization design process. Using the widely applied block model as the basis, a set of optimal production nested pits is obtained, allowing the realm design results to fit the coal price fluctuation trend and further enhance enterprise efficiency. The applicability and effectiveness of this method were verified by taking an ideal two-dimensional model and an inclined coal seam open-pit coal mine in Xinjiang as an example.

## Introduction

Open-pit mining method involves drilling layers of earth to expose ore and minerals while iteratively deepening the pit^1^. Excavated material from ore and waste rock pits is loaded onto trucks and transported to specific designated areas^2^. Open-pit mining is a complex system engineering, which primarily encompasses delineation of the mining boundary, engineering organization, and progress design. The size (or extent) as well as the sequence (or scheduling) of extraction are the most critical decisions within the domain of strategic or long-term production planning for open pit mining operations^3^. Once the boundary has been defined, the design of the deposit and the mining schedule are based on the mine's economic and technical capabilities. The essence of open-pit mining production is to extract the economic value behind mineral resources, which requires representing real-world deposits as idealized models with predetermined economic values. In economic modeling research, the most critical factor is the ore grade cut-off. Any blocks with grades below the cut-off are considered waste and only add to extraction costs. Conversely, blocks with grades above the cut-off are considered ore, generating revenue to offset extraction costs^4^. Three-dimensional block models are commonly employed as the primary methodology for estimating mineral resources and reserves within a mineral deposit^5^. There are three issues with the current method of defining production pits by setting a predetermined price for fixed-grade boundary: (1) assuming a fixed-grade boundary; (2) using nominal (monotonic increasing) prices to determine nested pits; and (3) neglecting the temporal interplay of resource requirements^6^. However, in the process of three-dimensional representation of mineral deposits, uncertainty from commodity prices and geological aspects always exists^7^, inevitably impacting the rationality of mining boundaries. Geological uncertainties persistently exist, which can impact the optimization results of mining boundaries. Changes in coal prices indirectly reflect the supply and demand relationship in the coal market, and thereby regulate the production capacity of coal enterprises^8^: (1) when coal prices rise, coal enterprises tend to expand production scale to improve their profitability; (2) when coal prices decline, enterprises tend to reduce production scale to mitigate potential economic losses. Adjusting mining boundaries is the simplest and most effective strategy for regulating production capacity in open-pit mining. Therefore, among the many factors affecting boundary optimization, changes in ore sales prices are particularly significant^9^. Based on the above description, coal prices are chosen as an example to illustrate the impact of variable indicators on boundary optimization design. The research findings can be easily extended to the study of other indicators on boundary optimization.

As a commodity, the price of coal is greatly affected by the external environment, which is directly reflected in its strong volatility. Hence, scientific and accurate coal price prediction assists relevant enterprises to anticipate the market trend and to prudently adjust economic activities^10^. Coal price forecasting can be categorized as an onerous time series modelling task. Different from ordinary regression, coal price forecasting needs to utilize multiple input characteristics of the coal price in a past period to predict its future trends^11^. Coal price forecasting methods in the literature can be roughly divided into two categories, namely, statistical models and artificial intelligence (AI) models. The former is based on conventional econometrics. Representative models primarily include vector autoregression (VAR)^12^, autoregressive integrated moving average (ARIMA)^13^, and generalized autoregressive conditional heteroskedasticity (GARCH)^14^. In recent years, thanks to the improvement of related algorithms and computing techniques, AI models have achieved major technological breakthroughs in energy price forecasting. The earliest recommended AI models are machine learning algorithms such as support vector regression (SVR)^15^, random forest (RF)^16^, and artificial neural networks (ANN)^17^. Machine learning methods make use of training data to better model the nonlinear mapping relationship between coal prices and their influencing factors^18^, which can improve the accuracy of forecasting results when predicting future price trends.

For open-pit mining operations, adjustments in production capacity due to changes in economic indicators require a certain level of buffer, as production capacity is not easily altered and adjustments are typically made only when indicators deviate beyond a certain range. This necessitates the consideration of a certain level of redundancy in the process, and the SVM method has been identified as an effective approach for addressing this issue. Meantime, the least squares support vector machine (LSSVM) method employs linear equality constraints instead of quadratic programming to solve complex problems, resulting in improved model performance^19^. As a typical machine learning method, SVM method is calibrated utilizing a structural risk minimization principle to minimize an upper bound of the generalization error^20^. The SVR method structure is more straightforward than fuzzy and ANN models that enhance the prediction model^21^. However, there has some unknown parameters in SVR structure, which drastically affect the prediction accuracy and generalization performance. Therefore, optimization of the model parameters is required to obtain a prediction model with good performance^22^. Many scholars have used metaheuristic algorithms for model parameter optimization due to their simple structure, fast efficiency, and few adjustment parameters^23^. The swarm intelligence optimization algorithms, inspired by collective behaviors of insects and other natural populations, are characterized by their high adaptability due to the absence of constraints and requirements on the nature and scale of the problem. As a result, these algorithms are widely applicable in various fields.

The sparrow search algorithm (SSA) is a new metaheuristic algorithm proposed by Xue and Shen^24^. Sparrow search algorithms have gained much attention because of its high efficiency, high convergence accuracy, and strong stability. Meantime, many scholars have improved SSA by increasing the initial population diversity and escaping from local optima. The tent chaotic mapping is used to initialize the population so that the initial individuals are distributed as evenly as possible^25^ while introducing Gaussian variation and chaotic perturbation to help individuals jump out of local optima, thus overcoming the drawback that SSA is prone to fall into local optima. Mao et al.^26^ integrated the idea of the sine–cosine algorithm to balance the local and global searchability in the discoverer position update method of SSA and introduced the Lévy flight strategy in the follower position update method to perturb the variation of the current optimal solution and strengthen the local escape ability, which obviously improved the efficiency of the SSA. Tang et al.^27^ used cubic mapping to initialize the population to obtain an improved chaotic sparrow search algorithm while using the Gaussian wandering strategy to help the algorithm jump out of stagnation.

Support Vector Regression (SVR) is capable of classifying the input space and obtaining a predictor without the need for data preprocessing, enabling quick understanding of the data's position in the space and greatly reducing computational requirements, particularly for complex data. However, the accuracy of SVR predictions relies heavily on the proper determination of kernel function parameters, as selecting incorrect parameters can easily result in inadequate prediction accuracy. The improved sparrow algorithm has achieved a good balance between computational efficiency and optimization performance, making it a viable option for solving complex problems. Based on this, the author believes that the LSSVM regression method using intelligent swarm-based decision-making algorithms, such as the sparrow search algorithm, holds certain advantages for coal price prediction. Based on the improved sparrow search algorithm (ISSA) proposed by Lv et al.^25^, a hybrid model of ISSA-based LSSVM (ISSA–LSSVM) was proposed for coal price forecasting. The optimal values of the penalty factor and kernel function parameter of the LSSVR model are selected by ISSA, and the model prediction results are applied to the prediction of coal price. Based on the research on the trend of coal prices, timely adjustment of open-pit mining production capacity to increase profits is theoretically feasible. According to neoclassical economics, producers always determine current output based on previous prices, and in a state of supply–demand equilibrium, current output will also affect current prices. Based on the above analysis, it is believed that coal prices have a regulatory mechanism on the production capacity of open-pit mines, i.e., changes in coal prices will guide the adjustment of production capacity by coal mining enterprises. When coal prices rise, enterprises will increase production capacity to obtain higher profits; when coal prices fall, enterprises will reduce production capacity to cope with potential losses. For open-pit mines, the simplest strategy to adjust production capacity is to adjust mining boundaries, which can be done relatively easily by modifying the length of working lines and advancement progress to make adjustments to the production range and thus control production capacity. Therefore, it is feasible to utilize the changing trend of coal prices as a basis for adjusting the boundary. This approach can enable the boundary to exhibit a similar changing trend as coal prices, thereby achieving the goal of improving enterprise efficiency and reducing risks.

The rest of the article is organized as follows: the basic principles of the LSSVM and ISSA methods are presented, and the hybrid ISSA–LSSVM model is briefly illustrated. The details of the case study and data and developed forecast models are then introduced before the statistics of the forecast results are presented and the results are discussed. Taking an ideal two-dimensional model and an open pit mine of an inclined coal seam located in Xinjiang, China, as an example, the applicability and effectiveness of the proposed method are verified. Finally, a discussion and conclusion are given, and the effectiveness and shortcomings of the proposed method are discussed.

## Improvement of the Sparrow search algorithm ISSA

Based on the research of relevant time series forecasting methods, the author believes that the improved support vector machine regression method based on intelligent group decision algorithms (such as the sparrow search algorithm) has certain advantages in coal price forecasting. However, the classical sparrow search algorithm has limitations such as the potential to fall into local optima. Therefore, it is necessary to improve the algorithm in order to further enhance prediction accuracy. The following section will provide a detailed discussion on this issue.

### Classic Sparrow Search Algorithm

**Figure 1:**
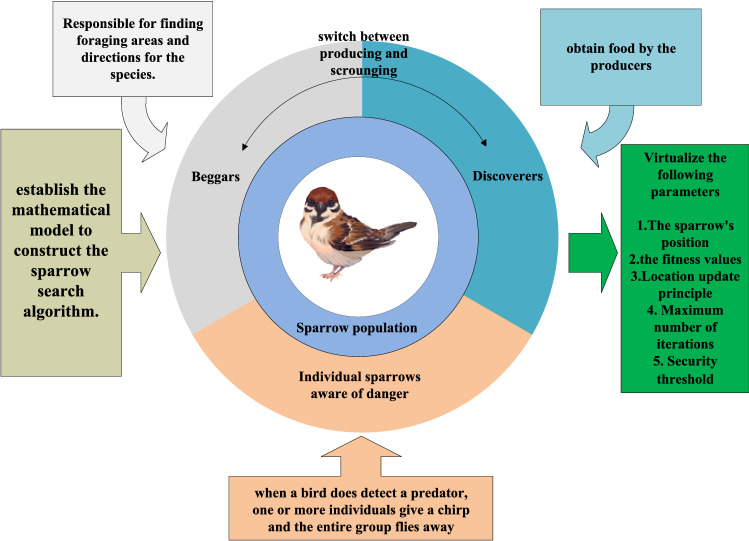
Schematic diagram of sparrow population division of labour.

SSA was proposed by Xue^24^ and primarily motivated by the foraging and anti-predator behaviors observed in sparrows. Based on assumptions about sparrow behavior, a virtual sparrow is used as the fundamental unit of the algorithm, where the aggregated positions of sparrows represent the optimal direction of the algorithm. The fitness function value is used as a substitute for sparrow energy reserves, which is then used to distinguish the roles played by sparrows within the population. Sparrows are known to prefer living in groups and have strong memories. They exhibit a clear division of labor within the population, with some individuals, known as “discoverers”, responsible for searching for food sources and providing information to the rest of the group, known as “beggars”, who obtain food based on the information provided by the finders. When there is a perceived threat from predators in the vicinity, a subset of sparrows within the population (typically selected randomly, unrelated to their role as finders or joiners) will emit alarm signals, triggering the entire population to engage in anti-predator behaviors to evade danger. The typical division of labor in a sparrow population is illustrated in Fig. 1.

Using virtual sparrows to find food by idealizing their behaviour. The sparrow's position can be represented by the following matrix.

1 $$X = \left[ {\begin{array}{*{20}c} {\mathop X\nolimits_{1} } \\ {\mathop X\nolimits_{2} } \\ \vdots \\ {\mathop X\nolimits_{N} } \\ \end{array} } \right] = \left[ {\begin{array}{*{20}c} {x_{11} } & {x_{12} } & \cdots & {x_{1d} } \\ {x_{21} } & {x_{22} } & \cdots & {x_{2d} } \\ \vdots & \vdots & \ddots & \vdots \\ {x_{n1} } & {x_{n2} } & \cdots & {x_{nd} } \\ \end{array} } \right]$$

where *n* is the number of sparrows and *d* is the dimension of the variable to be optimized. Then the fitness values of all individuals can be represented by the following vectors.

2 $$F_{X} = \left[ {\begin{array}{*{20}c} {\mathop f\nolimits_{1} } \\ {\mathop f\nolimits_{2} } \\ \vdots \\ {\mathop f\nolimits_{N} } \\ \end{array} } \right] = \left[ {\begin{array}{*{20}c} {f\left( {\left[ {\begin{array}{*{20}c} {x_{11} } & {x_{12} } & {\begin{array}{*{20}c} \cdots & {x_{1d} } \\ \end{array} } \\ \end{array} } \right]} \right)} \\ {f\left( {\left[ {\begin{array}{*{20}c} {x_{21} } & {x_{22} } & {\begin{array}{*{20}c} \cdots & {x_{2d} } \\ \end{array} } \\ \end{array} } \right]} \right)} \\ \vdots \\ {f\left( {\left[ {\begin{array}{*{20}c} {x_{n1} } & {x_{n2} } & {\begin{array}{*{20}c} \cdots & {x_{nd} } \\ \end{array} } \\ \end{array} } \right]} \right)} \\ \end{array} } \right]$$

The producers usually have high levels of energy reserves and provide foraging areas or directions for all joiners. They are responsible for identifying areas where rich food sources can be found. The level of energy reserve depends on the assessment of the fitness value of the individual. Producers with higher fitness values obtain the food first in the search process. In addition, producers are responsible for finding food and directing the movement of the entire population. Therefore, producers can search for food in a wider range of places than joiners. During each iteration, a producer's location is updated by the formula

3 $$x_{ij}^{t + 1} = \left\{ {\begin{array}{*{20}l} {x_{ij}^{t} \cdot \exp \left( {\frac{ - i}{{\alpha \cdot T_{max} }}} \right)} \hfill & {if\;R_{2} < ST} \hfill \\ {x_{ij}^{t} + Q \cdot L} \hfill & {if\;R_{2} \ge ST} \hfill \\ \end{array} } \right.$$

where *t* denotes the current iteration and *j* = 1, 2,…, *d*. $x_{ij}^{t}$ denotes the value of the *j*-th dimension of the *i*-th sparrow at iteration *t*. *T*_*max*_ is a constant representing the maximum number of iterations. *α* ∈ (0,1] is a random number. *R*_2_(*R*_2_ ∈ [0,1]) and *ST*(*ST* ∈ [0.5,1.0]) denote the alarm value and the safety threshold, respectively. *Q* is a random number that obeys a normal distribution. *L* denotes a matrix of order 1 × *d*. where each element is 1. When *R*_2_ < *ST*, indicating that there are no predators around, the producer enters the wide-area search mode. If *R*_2_ ≥ *ST*, some sparrows have found predators, and all sparrows need to fly quickly to other safe areas.

Sparrows with higher energy levels will act as producers. A few hungry joiners are more likely to fly elsewhere for more energy. Participants seek out food by following the producers who provide the best food. In addition, some subscribers may constantly monitor producers and compete for food to increase their own predation rates. The position update formula of a subscriber is

4 $$x_{ij}^{t + 1} = \left\{ {\begin{array}{*{20}l} {Q \cdot \exp \left( {\frac{{\mathop x\nolimits_{worst}^{t} - x_{ij}^{t} }}{{\mathop i\nolimits^{2} }}} \right)} \hfill & {if\;i \ge n/2} \hfill \\ {x_{p}^{t + 1} + \left| {x_{ij}^{t} - x_{p}^{t + 1} } \right| \cdot \mathop A\nolimits^{ + } \cdot L} \hfill & {otherwise} \hfill \\ \end{array} } \right.$$

The where *x*_*P*_ is the optimal position occupied by the producer. *x*_*worst*_ denotes the current global worst position. *A* denotes a 1 × *d* matrix where each element is randomly assigned to be 1 or − 1 and *A*^+^ = *A*^T^(*AA*^*T*^)^−1^. When *i* > *n*/2, this indicates that the *i*-th accession with a worse fitness value is most likely not to be allocated food.

Assuming that the number of sparrows aware of danger is 10–20% of the total population and that the initial positions of the above sparrows are generated randomly in the population, sparrows at the edge of the group will move quickly to a safe area to gain a better position when they are aware of danger, while sparrows in the middle of the group walk randomly to get closer to others, the mathematical model can be expressed as

5 $$x_{ij}^{t + 1} = \left\{ {\begin{array}{*{20}l} {\mathop x\nolimits_{best}^{t} + \beta \cdot \left| {x_{ij}^{t} - x_{best}^{t} } \right|} \hfill & {if\;f_{i} \ge f_{g} } \hfill \\ {x_{ij}^{t} + K \cdot \left( {\frac{{\left| {x_{ij}^{t} - x_{worst}^{t} } \right| }}{{\left( {f_{i} - f_{w} } \right) + \varepsilon }}} \right)} \hfill & {if\;f_{i} = f_{g} } \hfill \\ \end{array} } \right.$$

where *x*_*best*_ is the current global optimal position. The random number *β* ~ *N*(0,1) is the step control parameter. $K \in \left[ { - 1,1} \right]$ is a random number. *f*_*i*_ is the current fitness value of the sparrow. *f*_*g*_ and *f*_*w*_ are the current global best and worst fitness values, respectively. *ε* is the minimum constant to avoid zero division error. When *f*_*i*_ ≥ *f*_*g*_, the sparrow is at the edge of the population and vulnerable to predators; when *f*_*i*_ = *f*_*g*_, the sparrow is in the middle of the population. Once a sparrow is aware of a predator threat, it will approach other sparrows and adjust its search strategy to avoid being attacked.

### Improved Sparrow Search Algorithm

Despite the benefits of rapid convergence, high stability, minimal parameter tuning, and computational simplicity, the SSA algorithm may encounter the issue of local optima due to the random generation of individuals. Hence, this study proposes modifications to the SSA algorithm. Among the potential enhancements to various sparrow-inspired algorithms, considering the better local search ability of the Gaussian distribution and the characteristics of uniform traversal and fast convergence of the tent chaotic sequence, this article uses the tent chaotic mapping to initialize the population, make the initial individuals as uniformly distributed as possible, and introduce Gaussian variation and chaotic perturbations when the population appears to “gather” or “diverge”. The tent chaotic mapping is used to initialize the population so that the initial individuals are as evenly distributed as possible.

### Tent chaotic mapping

Chaos, as a nonlinear phenomenon prevalent in nature, has been applied to optimization search problems by many scholars because of the randomness, ergodicity and regularity of chaotic variables, which can not only effectively maintain the diversity of the population but also help the algorithm to obtain the global optimal solution and effectively prevent local convergence. Common logistic mappings have a high probability of taking values in the ranges [0, 0.05] and [0.9, 1], so the algorithm's optimization search speed is affected by the unevenness of the logistic traversal, and the optimization search efficiency will be reduced. (1) The study shows that the traversal uniformity and convergence speed of the tent mapping are better than those of the logistic mapping. Meanwhile, to avoid falling into small or unstable periodic points without destroying the three abovementioned characteristics of chaotic variables, introduced random variables rand(0,1) × 1/*N*_*T*_ into the original expression of tent mapping, and the improved expression is

6 $$z_{i + 1} = \left\{ {\begin{array}{*{20}l} {2z_{i} + rand\left( {0,1} \right) \cdot \frac{1}{{\mathop N\nolimits_{T} }}} \hfill & {0 \le z < \frac{1}{2}} \hfill \\ {2\left( {1 - z_{i} } \right) + rand\left( {0,1} \right) \cdot \frac{1}{{\mathop N\nolimits_{T} }}} \hfill & {\frac{1}{2} \le z \le 1} \hfill \\ \end{array} } \right.$$

The expression after the Bernoulli transformation is

7 $$z_{i + 1} = \left( {2z_{i} } \right)modl + rand\left( {0,1} \right) \cdot \frac{1}{{\mathop N\nolimits_{T} }}$$

where *N*_*T*_ is the number of particles within the chaotic sequence and rand(0,1) is a random number between [0,1].

According to the properties of the tent mapping, the steps to generate a chaotic sequence in the feasible domain are as follows:

- Step 1 Randomly generate the initial value *z*_0_ within (0,1), noting *i* = 0.
- Step 2 Iterate using Eq. (7) to produce a sequence of *Z* with *i* self-increasing by 1.
- Step 3 If the maximum number of iterations is reached, the program stops, and the resulting *Z* sequence is saved.

### Tent chaotic perturbations and Gaussian variation

A chaotic perturbation is introduced to prevent the algorithm from falling into a local optimum and to improve the global search capability and the accuracy of the search for an optimum. The steps of chaotic perturbation are described as follows.

- Step 1 Apply Eq. (7) to generate the chaotic variable *Z*_*d*_.
- Step 2 Carry the chaotic variables into the solution space of the problem to be solved:
  8 $$X_{new}^{d} = d_{min} + \left( {d_{max} - d_{min} } \right)Z_{d}$$
  where *d*_*min*_ and *d*_*max*_ are the minimum and maximum values of the *d*-dimensional variable $X_{new}^{d}$, respectively.
- Step 3 Add a chaotic perturbation to the individual according to Eq. (9):
  9 $$X^{\prime}_{new} = \left( {X^{\prime} + X_{new} } \right)/2$$
  where $X^{\prime}$ is the individual to be chaotically perturbed; $X_{new}$ is the amount of chaotic perturbation generated; and $X^{\prime}_{new}$ is the individual after the chaotic perturbation.

The Gaussian variation is derived from the Gaussian distribution, specifically when the variation operation is performed by replacing the original parameter value with a random number that fits a normal distribution with mean µ and variance σ^2^. The variance formula is

10 $$mutation\left( x \right) = x\left( {1 + N\left( {0,1} \right)} \right)$$

where* x* is the original parameter value; *N*(0,1) denotes a normally distributed random number with expectation 0 and standard deviation 1; and *mutation*(*x*) is the value after Gaussian variation. The Gaussian distribution has a strong local search capability, and for optimization problems with a large number of local minima, it is beneficial for the algorithm to find global minima with high efficiency and accuracy and improves the robustness of the algorithm.

## Implementation

**Figure 2:**
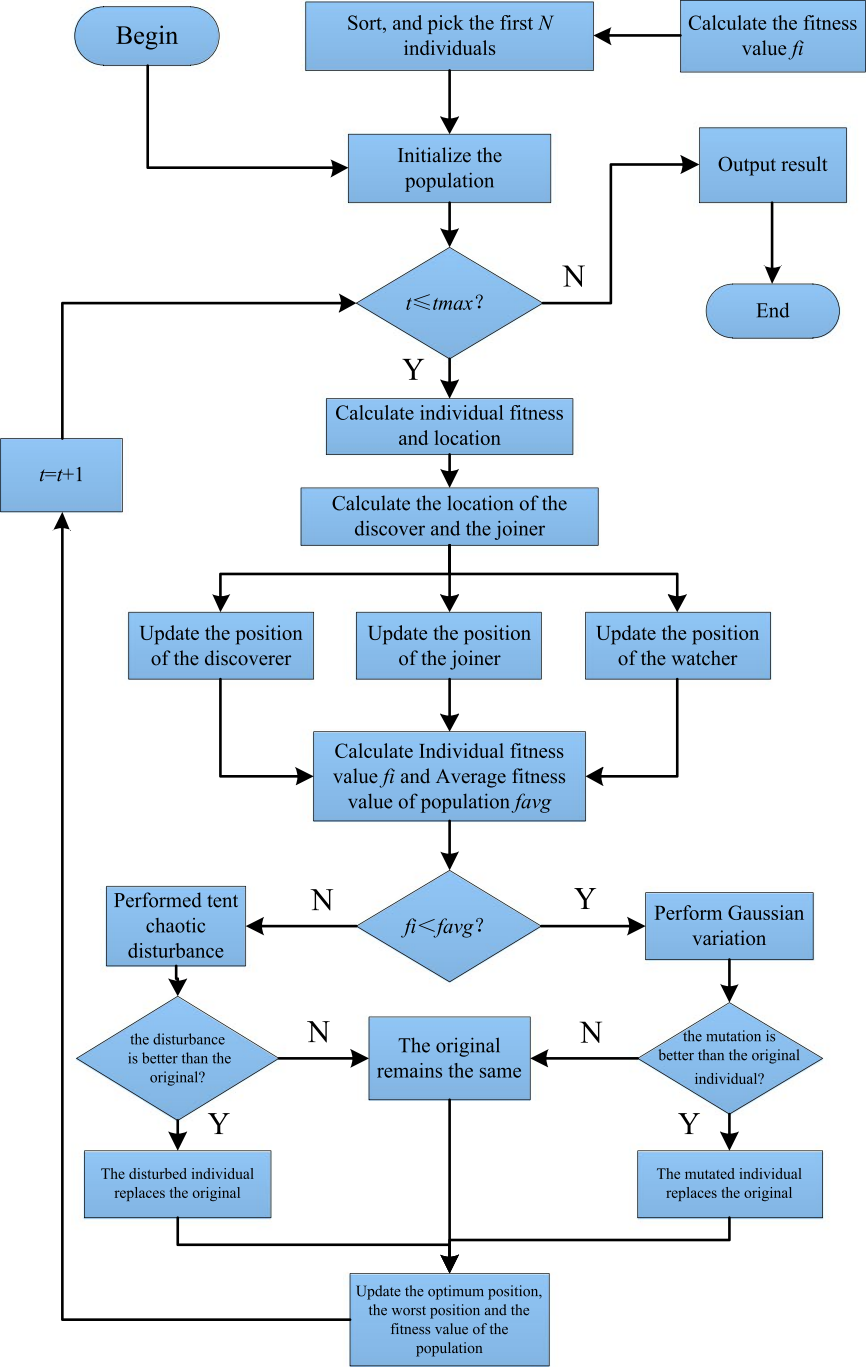
Flow chart of the ISSA.

Tent chaos search and Gaussian variation are introduced into the sparrow search algorithm, which is implemented as shown in Fig. 2.

The specific implementation steps are as follows:

- Step 1: Initialization, including population size, number of discoverers, number of scouts for alarm signals, dimensionality of the objective function, boundaries of initial values, maximum number of iterations, etc.
- Step 2: Initialize the population using Tent chaotic sequence, generating *D*-dimensional vectors with number *N*, and map the components to the range of variable values in the original problem space using Eq. (8).
- Step 3: Calculate the fitness of each sparrow, and identify the current best fitness value and its corresponding position, as well as the current worst fitness value and its corresponding position.
- Step 4: Select the top *p* fittest sparrows as discoverers, and the remaining sparrows as joiners, and update their positions using Eqs. (3) and (4).
- Step 5: Randomly select 10–20% of the sparrow individuals from the population as scouts for alarm signals, and update their positions using Eq. (5).
- Step 6: After each iteration, recalculate the fitness values of all sparrows and the average fitness value of the population.
  If *f*_*i*_ < *f*_*avg*_, indicating the occurrence of “clustering” phenomenon, apply Gaussian mutation. If the mutated individual is better than the original one, replace the original individual with the mutated one, otherwise keep the original individual unchanged.
  If *f*_*i*_ ≥ *f*_*avg*_, indicating the occurrence of “divergence” tendency, apply Tent chaotic perturbation to the individual. If the perturbed individual performs better than the original one, replace the original individual with the perturbed one, otherwise keep the original individual unchanged.
- Step 7: Update the best position and fitness value, as well as the worst position and fitness value, experienced by the entire population based on the current state of the sparrow population.
- Step 8: Check if the maximum number of iterations or desired solution accuracy has been reached. If so, terminate the loop and output the optimization result; otherwise, return to Step 4 for the next iteration.

## Least squares vector support machine method

**Figure 3:**
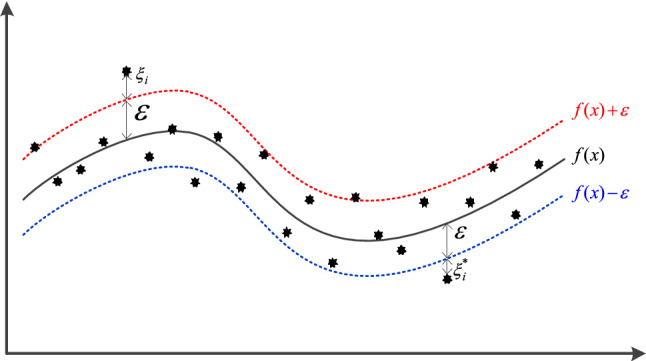
SVR 2D schematic.

SVR, as a branch derived from SVM, was proposed by Harris^28^. The output of SVR is discrete values, and by introducing the insensitive parameter *ε*, a new class of models with continuous values for the output is obtained, namely, SVR models. For prediction problems with small samples, the SVR model is used with good results, as shown in Fig. 3. SVR is a regression technique that aims to find a regression plane that minimizes the distances between a set of data points and the plane. Traditional regression methods typically consider predictions as correct only when the predicted value *f*(*x*) is exactly equal to the true value y, and commonly use (*f*(*x*) − *y*)/2 to calculate the loss. In contrast, SVR considers predictions to be accurate as long as the deviation between *f*(*x*) and *y* is within an acceptable range, and does not require explicit calculation of loss.

SVR is a quadratic programming (QP) problem, which introduces the concept of outliers into the support vectors and imposes inequality constraints, thereby increasing the difficulty in handling non-linear problems. Therefore, various improvement methods have been proposed, among which the Least Squares Support Vector Machine (LSSVM) is a modified version of traditional SVM that offers unique advantages in dealing with small sample sizes and non-linear problems. The main difference between LSSVM and SVM lies in the transformation of inequality constraints into equality constraints in LSSVM, which greatly simplifies the computation of Lagrange multipliers alpha and reduces the computational complexity, thereby improving the computational speed.

Given *m* training samples $D = \left\{ {\left( {x_{1} ,y_{1} } \right),\left( {x_{2} ,y_{2} } \right), \ldots ,\left( {x_{m} ,y_{m} } \right)} \right\} \, x_{i} \in R^{n} ,y_{i} \in R$, it is desirable to obtain a linear regression function $f\left( x \right) = w * \varphi \left( x \right) + b$ (where *ω is the* weight vector and *b* is the bias) such that it is as close as possible to the *y-*value at each point, with losses calculated only when $\left| {f\left( x \right) - y} \right| > \varepsilon$. This is equivalent to constructing an interval band of width 2*ε* with the original function *f*(*x*) as the median, and if the fitted value *y*_*i*_ falls into the interval band, it is considered to be a correct prediction and no loss is calculated. It can therefore be considered as providing some redundancy for the variation in the data during the time prediction process.

LSSVM can be used to map the input space to a high-dimensional feature space in time series regression with a nonlinear function that can be described as

11 $$\left\{ {\begin{array}{*{20}c} {\min \frac{1}{2}\left\| w \right\|^{2} + \frac{1}{2}C\mathop \sum \limits_{i = 1}^{m} \xi_{i}^{*} \mathop y\nolimits_{i} } \\ {s.t. \, \begin{array}{*{20}c} {y_{i} \left[ {\omega \cdot \Phi \left( {x_{i} } \right) + b} \right] = 1 - \xi_{i} } \\ {i = 1,2, \ldots ,m} \\ \end{array} } \\ \end{array} } \right.$$

where *C* > 0 is the penalty factor and $\xi_{i}$ is the error term.

To solve Eq. (11), the Lagrange operator is introduced, and the corresponding Lagrange function is constructed as

12 $$L\left( {\omega ,b,\xi_{i} ,\alpha } \right) = \frac{1}{2}\omega^{2} + \frac{1}{2}C\sum\limits_{i = 1}^{m} {\xi_{i}^{2} } - \sum\limits_{i = 1}^{m} {\alpha_{i} \left( {y_{i} \left( {\omega \cdot \phi \left( {x_{i} } \right) + b} \right) - 1 + \xi_{i} } \right)}$$

where *α*_*i*_ is the Lagrange multiplier.

The KKT condition gives the partial differentiation of in Eq. (12).

13 $$\left\{ {\begin{array}{*{20}c} {\frac{\partial L}{{\partial \omega }} = 0 \Rightarrow \omega - \mathop \sum \limits_{i = 1}^{m} \alpha_{i} \varphi \left( {x_{i} } \right) = 0} \\ {\frac{\partial L}{{\partial b}} = 0 \Rightarrow \mathop \sum \limits_{i = 1}^{m} \alpha_{i} = 0} \\ {\frac{\partial L}{{\partial \xi_{i} }} = 0 \Rightarrow C\xi_{i} - \alpha_{i} = 0} \\ {\frac{\partial L}{{\partial \alpha_{i} }} = 0 \Rightarrow \omega \cdot \varphi \left( {x_{i} } \right) + b + \xi_{i} - y_{i} = 0} \\ \end{array} } \right.$$

This can be simplified by eliminating $\omega$ and $\xi_{i}$ as follows

14 $$\left[ {\begin{array}{*{20}c} 0 & {e^{T} } \\ e & {\Omega + \frac{1}{\gamma }E} \\ \end{array} } \right]\left[ {\begin{array}{*{20}c} b \\ a \\ \end{array} } \right] = \left[ {\begin{array}{*{20}c} 0 \\ y \\ \end{array} } \right]$$

where: $$e^{T} = \left[ {1, \ldots ,L} \right]^{T}$$, $$y = \left[ {\mathop y\nolimits_{1} , \ldots ,\mathop y\nolimits_{m} } \right]$$, $$\Omega_{ij} = K\left( {x_{i} \cdot x_{j} } \right) = \varphi \left( {x_{i} } \right)^{T} \varphi \left( {x_{j} } \right),i = 1,2, \ldots ,m$$.

The role of kernel functions $K\left( {x_{i} \cdot x_{j} } \right)$ in SVR is to be able to map sample data from a low-dimensional space to a high-dimensional space without guaranteeing an increase in its inner product operations, enabling the process of processing nonlinear samples to linearise. Several common types of kernel functions are linear kernel functions, polynomial kernel functions, Gaussian radial basis (RBF) kernel functions, and multilayer perceptron kernel functions.

The selection of the kernel function is crucial to the effectiveness of the support vector machine model. The most widely used function in this paper is the Gaussian radial basis kernel function, whose expression is shown in Eq. (15).

15 $$K\left( {x_{i} \cdot x_{j} } \right) = \exp \left( { - \frac{{x - x_{i}^{2} }}{{\mathop g\nolimits^{2} }}} \right)$$

where *g* > 0 is the bandwidth of the Gaussian kernel and the kernel function parameters.

LSSVM modelling requires the setting of two key parameters: the penalty factor *C* and the kernel function parameter *g*. If the value of *C* is large, LSSVM overlearns; if the value of *C* is small, LSSVM underlearns. If *g* is too large, the LSSVM model accuracy decreases; if *g* is too small, the LSSVM model generalization ability decreases. The magnitude of the above two parameters determines the accuracy and the amount of redundancy of the time series prediction and plays a crucial role in the prediction effect. In general, the selection of penalty factor *C* and kernel function parameter *g* is still mainly based on human judgement, which may lead to distinctly different results due to differences between decision-makers. Therefore, this article proposes to use the ISSA algorithm to predict reasonable *C* and g values for the SVR model and then back-calculate the insensitive parameter *ε* to determine the amount of redundancy in the prediction period to improve the prediction accuracy and generalization performance of the prediction model.

The procedure for parameter selection of LSSVM using the ISSA is outlined as follows.

1. Data collection and preprocessing of the raw data.
2. Initialization of the algorithm parameters.
3. Utilizing LSSVM to obtain short-term prediction values based on the data.
4. Calculating fitness values by comparing the actual values with the predicted values, and determining the optimal solution.
5. Generating a new population based on the individual best positions and the global best position.
6. Obtaining new prediction values using LSSVM.
7. Comparing the fitness values of the new population with the previous best solution. If the fitness value is smaller, updating the optimal position; otherwise, keeping it unchanged.
8. Generating a new population based on the two new optimal positions.
9. Checking if the termination condition for optimization is satisfied. If yes, the iteration terminates; otherwise, incrementing the iteration counter and returning to step 6.
10. Assigning the optimized parameters to LSSVM for prediction, and finally outputting the optimal solution and fitness function (prediction error).
  Note: ISSA algorithm is used for parameter selection of LSSVM in this procedure.

## ISSA–LSSVR coal price time series forecast

### Construction of the time series prediction model

**Figure 4:**
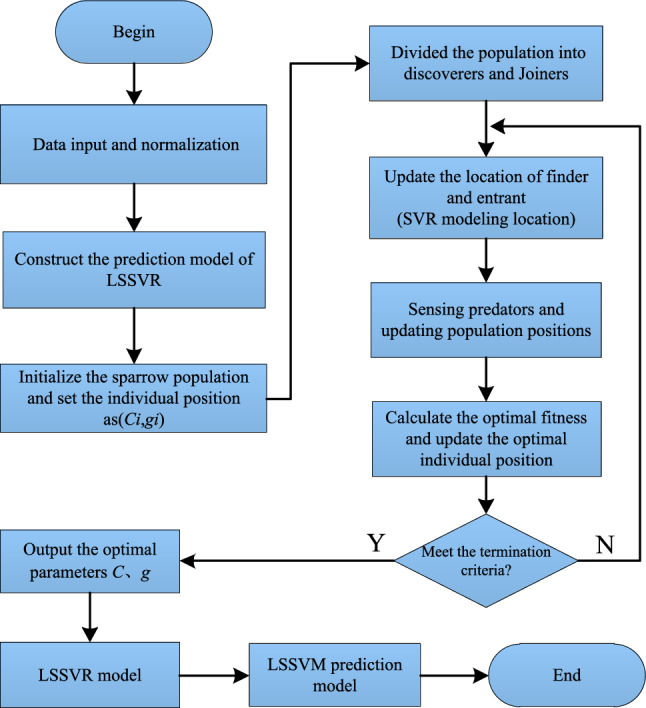
ISSA–LSSVR process.

In this article, we propose to use the LSSVR model for time series prediction. The performance of the LSSVR prediction model is mainly determined by the penalty factor *C* and the kernel function broadband *g*. Blind selection of parameters easily leads to the problems of low accuracy and low efficiency of the prediction model. ISSA has the advantages of high convergence accuracy, fast optimization and good stability. In this article, an ISSA–LSSVR prediction model is constructed, and the parameters of the LSSVR prediction model are optimized by using ISSA to search for the combination of penalty factor *C* and kernel function parameter *g*. The flow chart is shown in Fig. 4.

The pseudocode for the ISSA–LSSVM is presented in Algorithm 1.
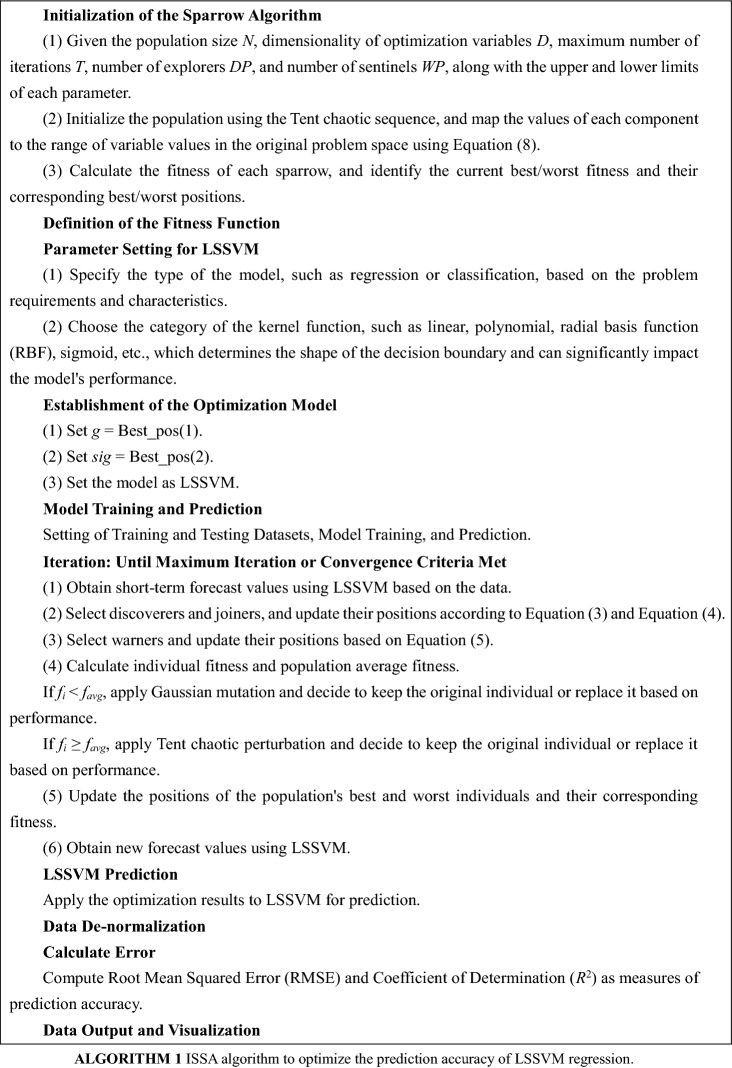


### Coal price data collection

**Figure 5:**
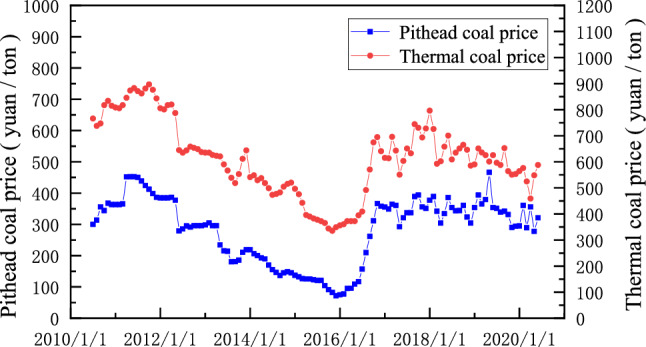
Comparison of coal pithead price and thermal coal price trend.

This article uses the Prospective Database website (https://d.qianzhan.com/) to collect raw coal prices and power coal prices for the 10-year period 2010–2020, the trends of which are compared in Fig. 5

As shown in Fig. 5, the trends of raw coal pit prices and the value of power coal show a strong correlation, i.e., the trends are essentially the same for both. For surface mines, where companies obtain their economic value through the direct sale of raw coal, the pithead price of coal is expected to significantly impact the production scale of open-pit mines. Therefore, the pit price is the focus of this article. Historical trends show that the pit price of raw coal has experienced a number of upwards and downwards movements, and the fluctuations up and down have been dramatic.

### Data processing and coal price forecasting

By collecting raw coal pithead prices over a ten-year period from July 2010 to June 2020, the daily raw coal prices were chosen to obtain monthly average prices to better reduce the error in price prediction. A total of 120 monthly coal price data points were selected, and the dataset was sliced in order, with the first 100 sets of data used as the training set and a fivefold cross-validation to ensure model training accuracy, and the last 20 sets of data used as the test set. The training set was used to train each model, and the test set was used to test and update the parameters of the model.

The sparrow population was selected as 30, and the maximum number of iterations was set as 50. The ISSA–LSSVR prediction model was used to predict the coal price time series according to the flow in Fig. 3. The most important parameters in the sparrow search algorithm are set as follows: the warning value is set to 0.6, the proportion of discoverers is 70% (the rest are entrants), and 20% of the individuals in the population are randomly selected and assumed to be aware of the danger. Tent chaotic mapping was used to initialize 3 population locations, and 30 individuals with the best fitness were selected as the initial population. The above parameters are the optimal values obtained by referring to a large number of ISSA algorithms, which effectively improve the running speed in the case of high precision.

**Figure 6:**
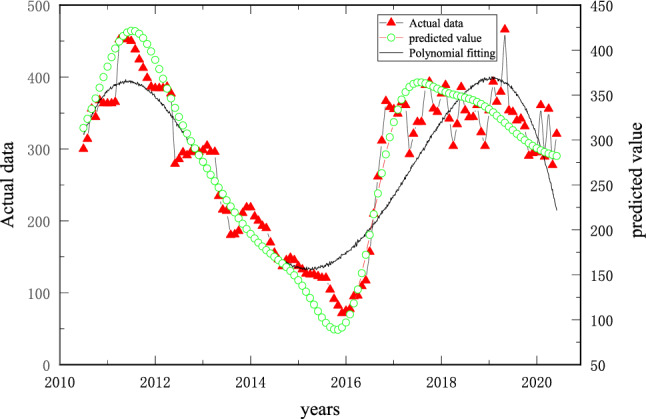
ISSA–SVR model prediction results.

**Figure 7:**
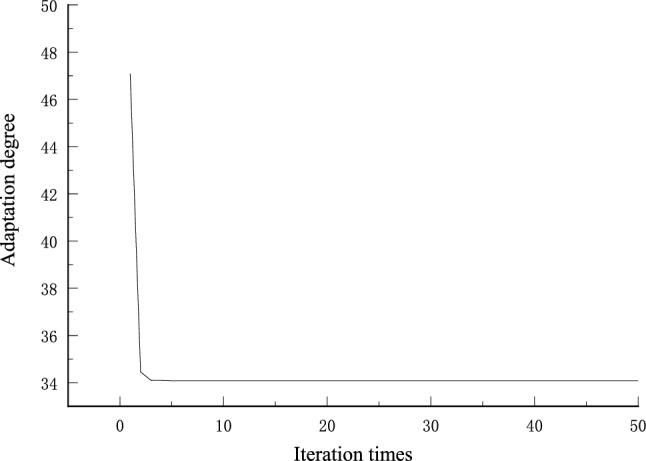
Plot of adaptation with number of iterations.

The sparrow algorithm operation results showed that the optimal penalty factor *C* = 1.9524, the kernel function parameter *g* = 0.1, and the deviation term is 0.054; that is, only the data exceeding the existing coal price by ± 5.4% are included in the error. The coal price prediction results are shown in Figs. 6 and 7.

Polynomial regression is used to fit the historical change trend of coal prices, as shown in Fig. 6. Data show that from 2010 to 2020, the coal price experienced four ups and downs. From the middle of 2010 to the end of 2011, the rapid development of China's social economy greatly stimulated the demand for coal and brought about the continuous growth of coal industry output and price. Coal prices also continued to rise in the shock, to the overall relatively highest state in history. Since 2012, economic development has levelled off, and the growth rate of energy consumption has slowed down rapidly. Affected by the impact of overcapacity caused by blind expansion, the coal industry entered a winter period, and the overall loss of the industry was large. In 2016, with the proposal and implementation of the national supply-side reform and other policies, the coal industry began to gradually recover, and thermal coal prices also began a two-year upwards shock, rising by more than 100%. In 2018, high-quality coal production capacity began to decrease, the overall coal supply turned loose, and thermal coal prices began to fall steadily from high levels. Until the beginning of 2020, thermal coal prices fell sharply due to the impact of COVID-19.

By comparing the fitting results of the fourth-order polynomial in Fig. 6 with the ISSA–SVR prediction method, the prediction method proposed in this paper obviously has higher prediction accuracy. It can be seen that the Sparrow algorithm can approach the optimal fitness value in three iterations, which illustrates the effectiveness of the ISSA algorithm; the sample fit of the training set is high, with *R*^2^ being 0.94247; the accuracy of the test set is poor, with *R*^2^ being 0.18223, indicating that the algorithm in the article is less capable of handling abnormal fluctuations in the data, but the prediction results can analyse the trend of coal price fluctuations and have certain application effects.

### Coal price forecasting model accuracy test and analysis of forecasting results

To illustrate the effect of the algorithm proposed in the article, a traditional time series forecasting model is selected for comparative analysis. The autoregressive moving average model (ARIMA) has been more widely used in the forecasting of price time series in recent years. The model is mainly controlled by three indicators: the number of autoregressive terms p, the number of moving average terms q and the number of differences d. That is, the ARIMA (*p*, *d*, *q*) model takes the form of

16 $$\varphi \left( L \right)\left( {1 - L} \right)^{d} X_{t} = \theta \left( L \right)\varepsilon_{t}$$

where $\varphi \left( L \right)$ and $\theta \left( L \right)$ are stationary lag operators of order *p* and order *q,* respectively; *L* is the lag operator, i.e., $LY_{t} = Y_{t - 1}$. {*ε*_*t*_} is the white noise sequence; *d* is the difference parameter; *c* is a constant; *p*, *d* and *q* are nonnegative integers.

**Figure 8:**
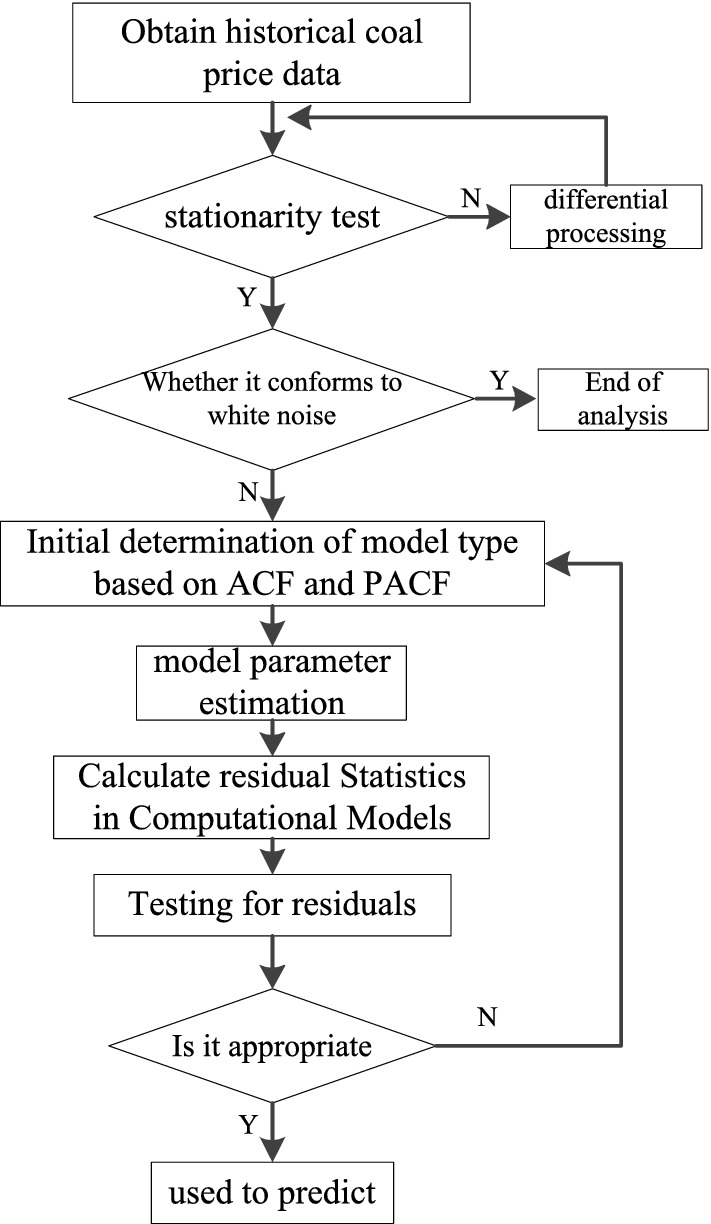
The ARIMA model prediction flow chart.

In general, the modelling process of the price series predicted by the ARIMA model is shown in Fig. 8.

**Figure 9:**
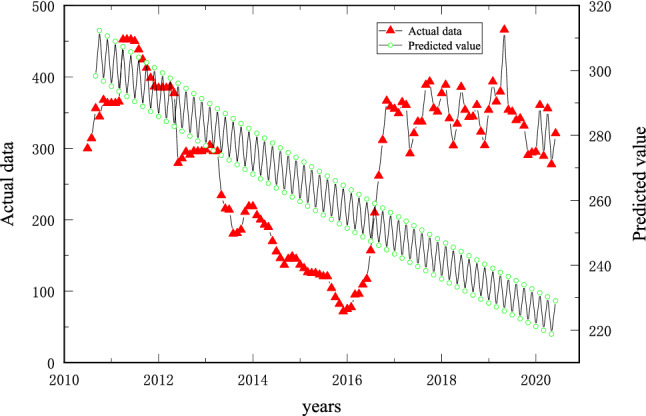
Coal price ARIMA model prediction results.

The stability analysis showed that the original data were a non-time smooth series, which was differenced, and the 1st order differenced series was a smooth time series. The model parameters were chosen as ARIMA(2,1,0). The prediction results of the ARIMA model are shown in Fig. 9.

Two indices, including the root mean square error (RMSE) and coefficient of determination (*R*^2^), were used to evaluate the validity of the prediction model. The formulas are as follows

17 $$RMSE = \sqrt {\mathop \sum \limits_{i = 1}^{N} \left( {\hat{y}_{i} - y_{i} } \right)^{2} }$$

18 $$R^{2} = 1 - \frac{{\mathop \sum \nolimits_{i = 1}^{N} \left( {y_{i} - \hat{y}_{i} } \right)^{2} }}{{\mathop \sum \nolimits_{i = 1}^{N} \left( {y_{i} - \overline{y}} \right)^{2} }}$$

A comparison of the ISSA–LSSVR model and the ARIMA model predictions is shown in Table 1.

**Table 1:** Comparison of predictive model effects.

Model categories	Training set		Test set	
	RMSE	*R*^2^	RMSE	*R*^2^
ISSA–LSSVR	26.7215	0.9425	50.2477	0.1822
ARIMA	108.3521	0.8648	117.5293	0.1236

From Fig. 6, Fig. 9 and Table 1, it can be seen that both the training set and the test set data show that the ISA-LSSVR model has good fitting accuracy. Meanwhile, due to the characteristics of the SVR model, certain redundancies can be considered in the process of prediction, which conforms to the idea of flexible optimization and optimizes the flexible interval to some extent.

## Optimal design of a real opencast mine under coal price fluctuations

### Uncertainty risk nesting properties

A disadvantage of traditional realm optimization methods is that the parameters used in the calculation of the economic value of a block are relatively fixed, and uncertainty cannot be measured effectively. To choose an appropriate risk measure for the above problem, it is necessary to define the ideal characteristics of the pit generated by the risk measure ρα when varying the risk aversion *α*. The idea of nesting can be applied to generate a set of nested pits containing ordered levels of risk, provided that the risk is managed.

Combining the constraints on constant normalisation and translation consistency to be satisfied by the risk metric with the nesting idea, the risk nesting property can be derived for the open-air optimal design process^29^.

1. Risk nestedness.
  Assuming that the blocks remain independently distributed within the mine area, as the parameter *α* increases, the risk measure *ρ*_*α*_ of the decision process increases, and the pit generated using the lower risk aversion level *α*_1_ should be fully contained within the pit generated using the higher risk aversion level *α*_2_.
2. Additive consistency.
  Suppose the random variable *Z* is independent of the intrinsic random variables *X* and *Y*, and the variable *ρ*_*α*_() represents the risk measure at risk aversion *α*. If $\rho_{\alpha (X)} < \rho_{\alpha (Y)} \to \rho_{\alpha (X + Z)} < \rho_{\alpha (Y + Z)}$, then *ρ*_*α*_() is said to satisfy additive consistency. That is, the addition of a certain number of blocks to the pit at different stages cannot change its original risk measure.

**Figure 10:**
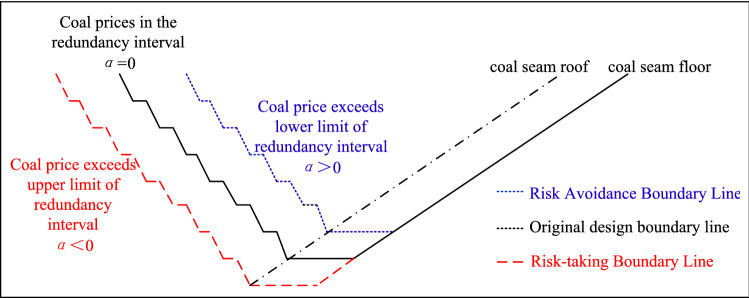
Schematic diagram of nested pits for the boundary optimization problem of open pit mines.

As shown in Fig. 10, when the production of an open pit mine is affected by uncertainties, the option of producing according to the original design boundary is questioned. A positive or negative value of *α* can be used to reflect the different risks faced by the open pit mine: when *α* < 0, the tendency is to take risks, and the open pit boundary needs to be expanded to reduce the company's lost profit; when α > 0, the tendency is to avoid risk, and the open pit boundary needs to be reduced. When α > 0, the tendency is to be risk-averse, and the open pit boundary should be reduced to minimize the loss of profit. Combined with the SVM method, an interval band with a width of 2*ε* is constructed with the preset coal price as the middle line: when the actual coal value is in the interval band, no loss is calculated, i.e., the value of α is considered not to change; when the actual coal price is outside the interval band, the value of α is considered to change, and the realm needs to be adjusted at this time.

### Optimization and adjustment of coal limit under the fluctuation of coal price

Usually, the greater the difference between the fixed coal price index and the actual coal price data, the greater the risk the enterprise will bear. As shown in the analysis of the coal price variation trend mentioned above, the coal price fluctuation trend is strong during the study period, and the change in coal price will have an impact on the economic effect of enterprises. The price of coal is primarily influenced by the coal market, and price fluctuations in coal can impact coal sales, thereby providing feedback to coal prices, thus forming a cyclical structure. In neoclassical economics, the cobweb model is used to investigate the interactions between demand, supply, and prices of products with longer production cycles. This theory proposes that producers always determine current output based on previous period prices, and in a state of supply and demand equilibrium, current output will in turn affect current prices. Therefore, it can be argued that changes in coal prices will have a positive impact on the production capacity of coal companies. If the trend of coal prices can be predicted in advance, adjustments to production capacity can be made proactively, leading to greater economic benefits. For open-pit coal mines, the simplest and most effective method of adjusting production capacity is to make adjustments to the mining boundary. If we can follow the trend of coal prices and adjust the boundary, we can improve the earnings of enterprises to a certain extent. In contrast, although the size of the limit will not affect the fluctuation of the coal price, its current status limits the range of limit adjustment. If the limit can be adjusted in advance, it may avoid situations in which the limit cannot be expanded/reduced in a timely manner due to the restriction of mining procedures.

**Figure 11:**
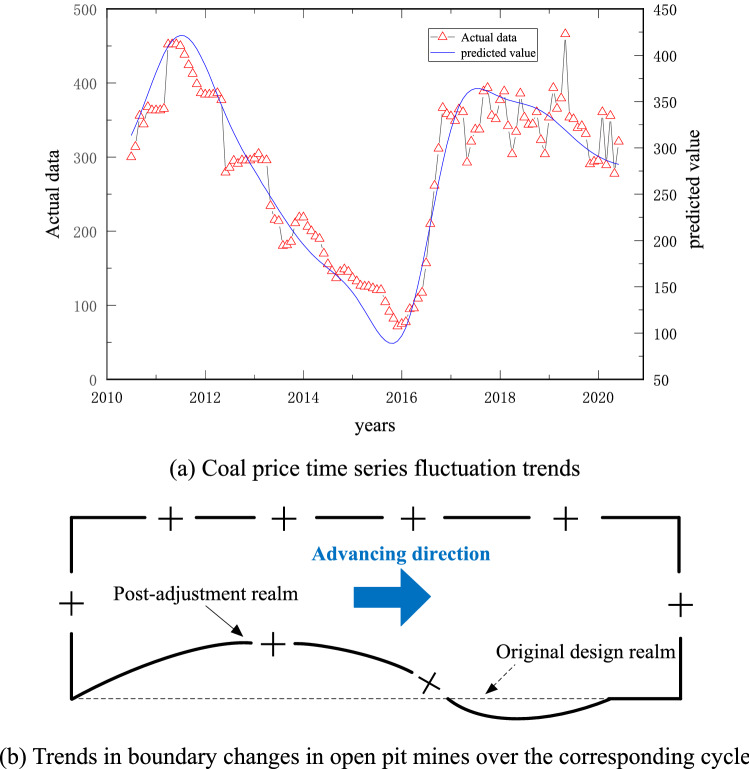
Comparison of the final state adjustment of open pit mines and coal price volatility.

The ISSA algorithm shows that when the fluctuation value of the coal price exceeds the existing price by ± 5.4%, the limit needs to be adjusted. In this case, the corresponding relationship between the final mining limit change and the coal price fluctuation is shown in Fig. 11.

The trend of the coal price series shows that the raw coal price has gone up and down four times, with an average price of 285.85 yuan, and the coal price is highly volatile. Dynamic adjustment of the final mining realm of the open pit based on coal price fluctuation trends can effectively address the risks arising from deviations of preset profit targets from actual scenarios and enhance corporate efficiency.

## Case study

To verify the effectiveness of the proposed method in solving the optimization problem of open pits under uncertain conditions, the two-dimensional topology of an ideal small open pit was selected as an example. The principle and process of open pit limit adjustment in inclined coal seams under the condition of coal price change are explained. Taking an open pit mine of an inclined coal seam in Xinjiang, China, as an example, the coal price variation trend obtained by the ISSA–SVR prediction method is applied to the limit adjustment of the open pit mine.

### Topology of an ideal small inclined coal seam opencast mine

**Figure 12:**
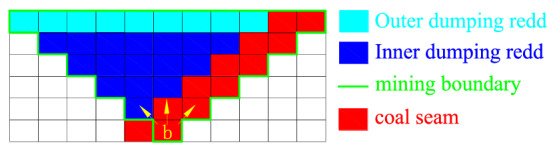
Two-dimensional topology structure of a small inclined coal seam open pit mine.

To ensure the validity of the model, it is assumed that the advancement of this opencast coal mine remains consistent across the range of years. The two-dimensional topology of this small inclined coal seam opencast mine is shown in Fig. 12.

It is assumed that the coal seam dip angle and final slope angle are both 45°. At the same time, the inner and outer stripping are distinguished, and the constraint of block extraction priority is given. It is assumed that the mining boundary of the open pit mine is variable when the external environment changes, that is, when the coal price exceeds the preset value, the mining depth can be appropriately increased to expand the open pit mine limit and raw coal output, and vice versa.

**Figure 13:**
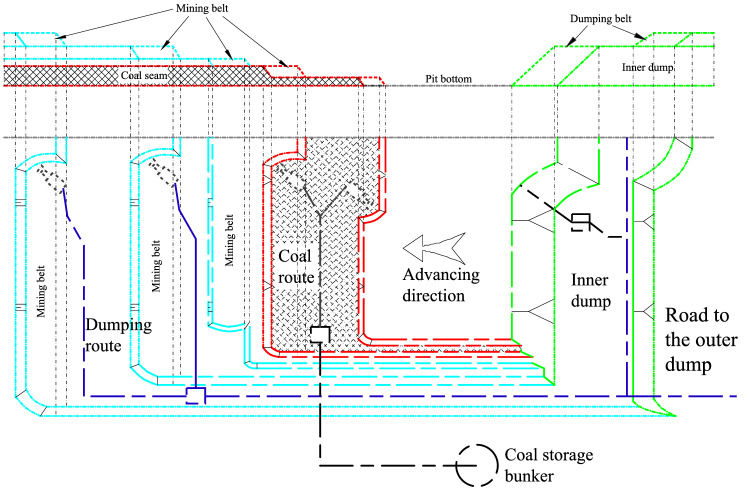
Schematic diagram of the layout of the open pit mine mining and stripping project.

For open pit coal mines, assume an a priori decision of what is ore (valuable) and waste material, which is commonly referred to as a cut-off grade policy^4^. The blocks mined within the limits generally only contain two destinations: the stripping material is transported to the dump for discarding through the transportation line, and the raw coal is transported to the coal storage yard for further treatment through the main transportation system of the mine. The layout diagram of open pit mining and stripping processes is shown in Fig. 13.

For all blocks within the mining scope, mining cost *c*_*b*_ is assigned. In addition, given the priority constraint of each block, it follows the constraint of the maximum side slope angle. To simplify the description process, *p*_*b*_ is taken as the random variable representing the profit of the block. It is assumed that the cost of mining the unit volume of the block is $c_{b}^{e}$, the unit transportation cost is $c_{b}^{t}$, the unit postprocessing cost of the block is $c_{b}^{p}$, the internal and external stripping distances are taken as *L*_*n*_ and *L*_*w,*_ respectively, and the raw coal distances are taken as *L*_*m*_. Based on the research on boundary grade by Ares et al.^5^, a formula for calculating the value of coal blocks in open-pit coal mines is proposed. Taking into account the characteristics of coal deposit occurrence, the formula for calculating the value of a coal block (*v*_*b*_) in open-pit mining is developed.

19 $$v_{b} = \left\{ {\begin{array}{*{20}c} {c_{b}^{e} + c_{b}^{t} \times L_{m} + \left( {c_{b}^{p} - r_{b} p_{b} } \right) ({\text{Ore block)}}} \\ {c_{b}^{e} + c_{b}^{t} \times L_{n} {\text{(Inner row waste block)}}} \\ {c_{b}^{e} + c_{b}^{t} \times L_{w} {\text{(Outer row waste block)}}} \\ \end{array} } \right.$$

where $p_{b}$ is the profit of the treated unit block (selling price of raw coal under deterministic conditions) and $r_{b} > 0$ compensates for other factors that affect block profits.

Let $P \subseteq B \times B$ be the priority constraint set, that is, for (*b*, *b*′) ∈ *P*, block *b′* must be mined before block *b*, and then the medium- and long-term boundary optimization problem is expressed with cost minimization:

20 $$\begin{gathered} UP = \mathop {\min }\limits_{{\mathop x\nolimits^{e} ,\mathop x\nolimits^{p} }} \mathop \sum \limits_{b \in B} (c_{b}^{e} x_{b}^{e} + c_{b}^{t} x_{b}^{n} \times L_{n} + c_{b}^{t} x_{b}^{w} \times L_{w} \hfill \\ + c_{b}^{t} \times L_{m} ) + \mathop \sum \limits_{b \in B} \left( {c_{b}^{p} - r_{b} p_{b} } \right)x_{b}^{p} \hfill \\ s.t. \hfill \\ x_{b}^{e} \le x_{{b^{,} }}^{e} ,\forall \left( {b,b^{,} } \right) \in P, \hfill \\ x_{b}^{e} ,x_{b}^{p} ,x_{b}^{n} ,x_{b}^{w} \in \left\{ {0,1} \right\},\forall b \in B, \hfill \\ \end{gathered}$$

where $x_{b}^{e} \le x_{{b^{,} }}^{e}$ is the block priority constraint; $x_{b}^{p}$ is the postprocessing decision; $x_{b}^{e}$ is the mining decision; $x_{b}^{n}$ is the internal scheduling decision; $x_{b}^{n}$ is the outgoing decision; and $x_{b}^{p}$, $x_{b}^{e}$, $x_{b}^{n}$, and $x_{b}^{w}$ are binary random variables whose values can be 0 or 1.

### Limit adjustment of small open pit mine under coal price fluctuation

Generally, the range of coal price change is small, so it is assumed that the range of coal price change is the current coal price ± 50%. At the same time, to ensure the continuity of the project, it is assumed that the change of the actual coal price caused by the change of the boundary is not more than 20% of the existing coal price, and different coal price fluctuation ranges correspond to different *α* values: (1) when the change of coal price is in the range of (− 5.4%, 5.4%), *α* is considered to be 0; (2) when the coal price changes are at [− 50%, − 5.4%] and [5.4%, 50%], the data are normalized and the corresponding *α* value is calculated, that is, an *α* value of ± 5.4% corresponds to 0, an α value of 50% corresponds to − 1, and an *α* value of − 50% corresponds to 1. The part exceeding ± 50% is calculated as ± 50%.

**Figure 14:**
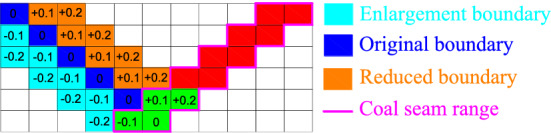
Schematic diagram of two-dimensional boundary adjustment under risk conditions.

The fluctuation cycle of coal prices in Fig. 6 is selected as the object of this study, and we refer to Fig. 10a. The coal price presents a combined trend of change, and the adjustment of the boundary should follow the change in the coal price. However, considering the range of the adjustment of the boundary, the length of the working line should be as close as possible to the length of the working line calculated from the actual coal price, as shown in Fig. 14.

As shown in Fig. 14, (1) when *α* = 0, it represents risk neutrality, that is, there is no deviation between the preset coal price index and the actual coal price, and there is no need to adjust the level at this time; (2) when *α* is + 0.1 and + 0.2, it means that the preset coal price of the open pit mine is higher than the actual coal price, and the enterprise needs to bear the risk of loss. In this case, it is necessary to reduce the open pit mine limit. (3) When *α* is − 0.1 and − 0.2, the preset coal price of the open pit mine is lower than the actual coal price. In this case, it is necessary to expand the open pit mine limit.

It is assumed that the small open pit coal mine is in a partial internal discharge period from 2010 to 2022, that is, a small part of the stripping is transported to the outer dump, and most of the stripping is transported to the inner dump for discharge. To simplify the description process, it is assumed that the top flat strip is transported to the outer dump and the remaining flat strip is internally discharged. The block in the mining limit contains three directions: inner and outer dump and coal storage bin. The main economic indicators of the production link of the open pit mine are given in Table 2.

**Table 2:** Main economic indicators in the production link.

Name of index	Unit	Numerical value
Cost of extraction	yuan m^−3^	10
Cost of transportation	yuan m^−3^ km^−1^	2
Post-processing cost	yuan m^−3^	5
Internal drainage distance	km	2
Outbound running distance	km	4
Raw coal transport distance	km	3
Preset coal profit	yuan m^−3^	− 60
Recovery of resources	%	95

**Figure 15:**
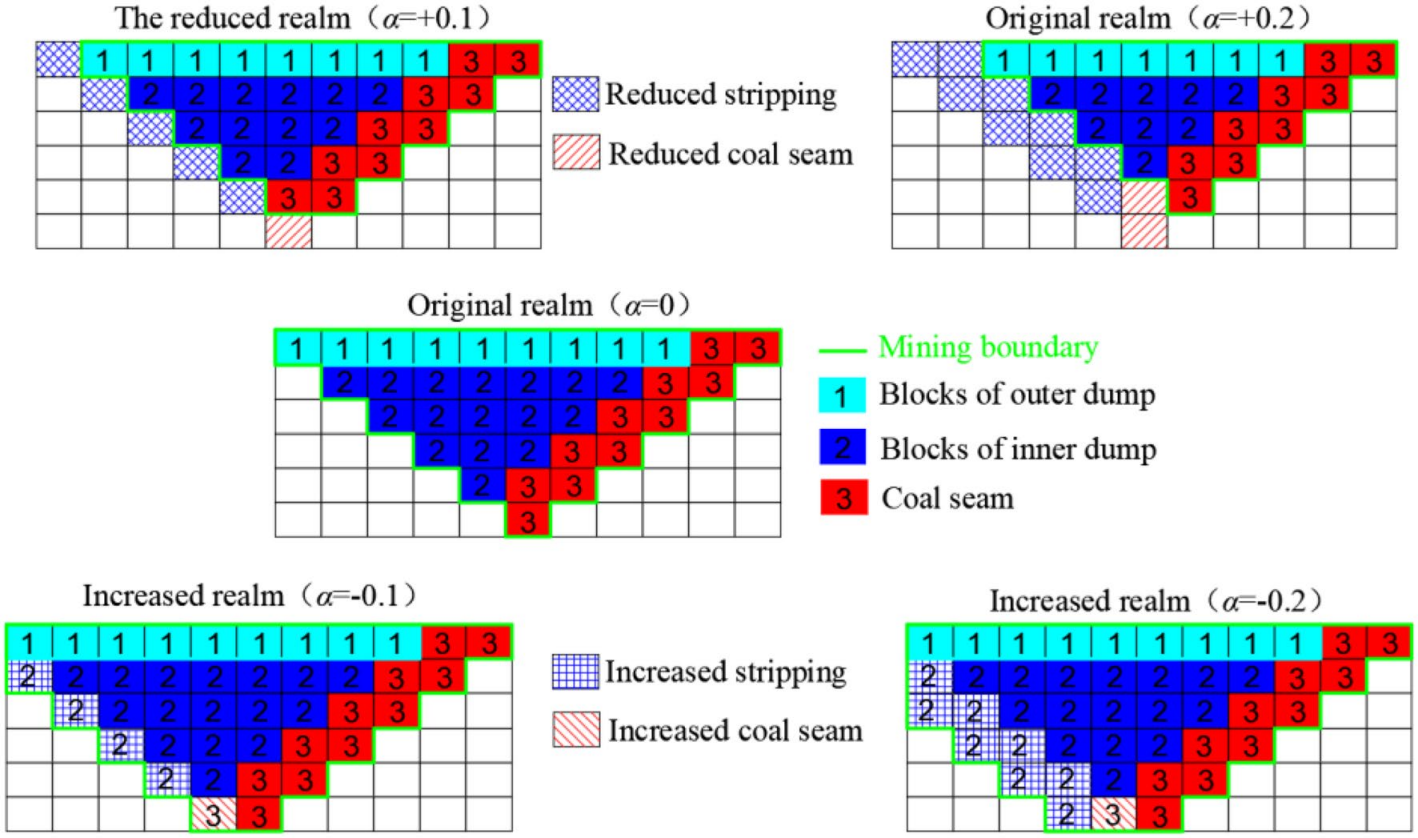
Schematic diagram of the block model section of a small open-pit mine.

The block model profile of a small open pit mine is shown in Fig. 15. The blocks labelled “1”, “2” and “3” are transported to the outer dump, the inner dump and the coal storage bin, respectively, assuming that the volume of the blocks is 1 m × 1 m × 1 m. Below, the profits in different mining limits are described in terms of different values of *α*.

1. *α* = 0
  At this time, there is no deviation between the actual coal price and the preset coal price; that is, the risk is neutral. According to Eq. (20), the net profit in the mine is *W*_0_ = 9 × (10 + 2 × 4) + 16 × (10 + 2 × 2) + 11 × (10 + 2 × 3 + 5 − 60 × 0.95) = − 10 yuan.
2. *α* < 0
  Considering that the expansion of the limit should be accompanied by a depth reduction of the open pit mine, the limit can be expanded only when the actual coal price exceeds 20% of the preset coal price, i.e., *α* = − 0.2. Taking the profit of raw coal as 67.6 yuan, according to Eq. (20), the net profit calculated is *W*_-0.2_ = 9 × (10 + 2 × 4) + 24 × (10 + 2 × 2) + 12 × (10 + 2 × 3 + 5 − 67.6 × 0.95) = − 20.4 yuan.
3. *α* > 0
  When the actual coal price is 10% lower than the preset coal price, *α* =  + 0.1, then the level can be reduced. Taking the profit of raw coal as 56.8 yuan, according to Eq. (20), the net profit calculated is *W*_+0.1_ = 8 × (10 + 2 × 4) + 12 × (10 + 2 × 2) + 10 × (10 + 2 × 3 + 5 − 56.8 × 0.95) = − 12 yuan.
  When the raw coal price increases by 20% and decreases by 10%, the adjustment of the open pit limit can increase the net profit of the pit by approximately 104% and 20%, respectively, and at the same time ensure that the risk is under control.

### A practical example of an inclined coal seam open pit mine

**Figure 16:**
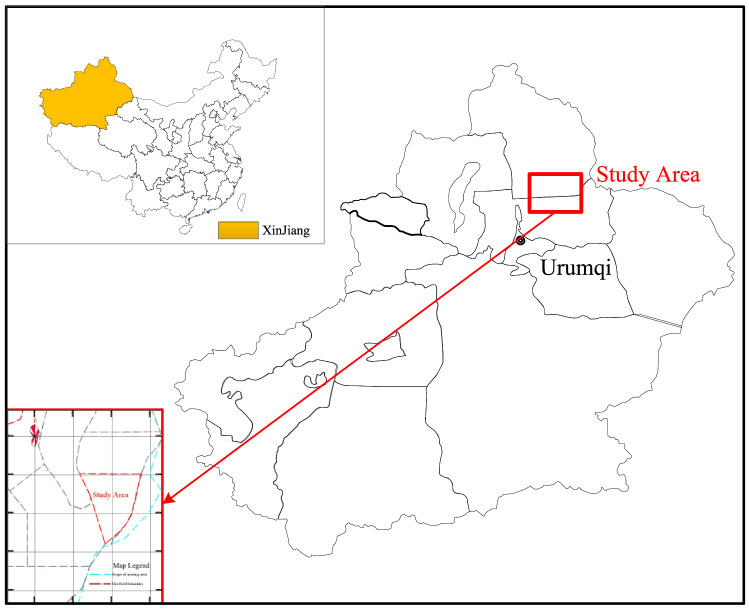
The location of the study area.

To illustrate the effectiveness of the algorithm in the present study, a case study was conducted in a selected open-pit coal mine located in Xinjiang, China, as depicted in Fig. 16.

**Figure 17:**
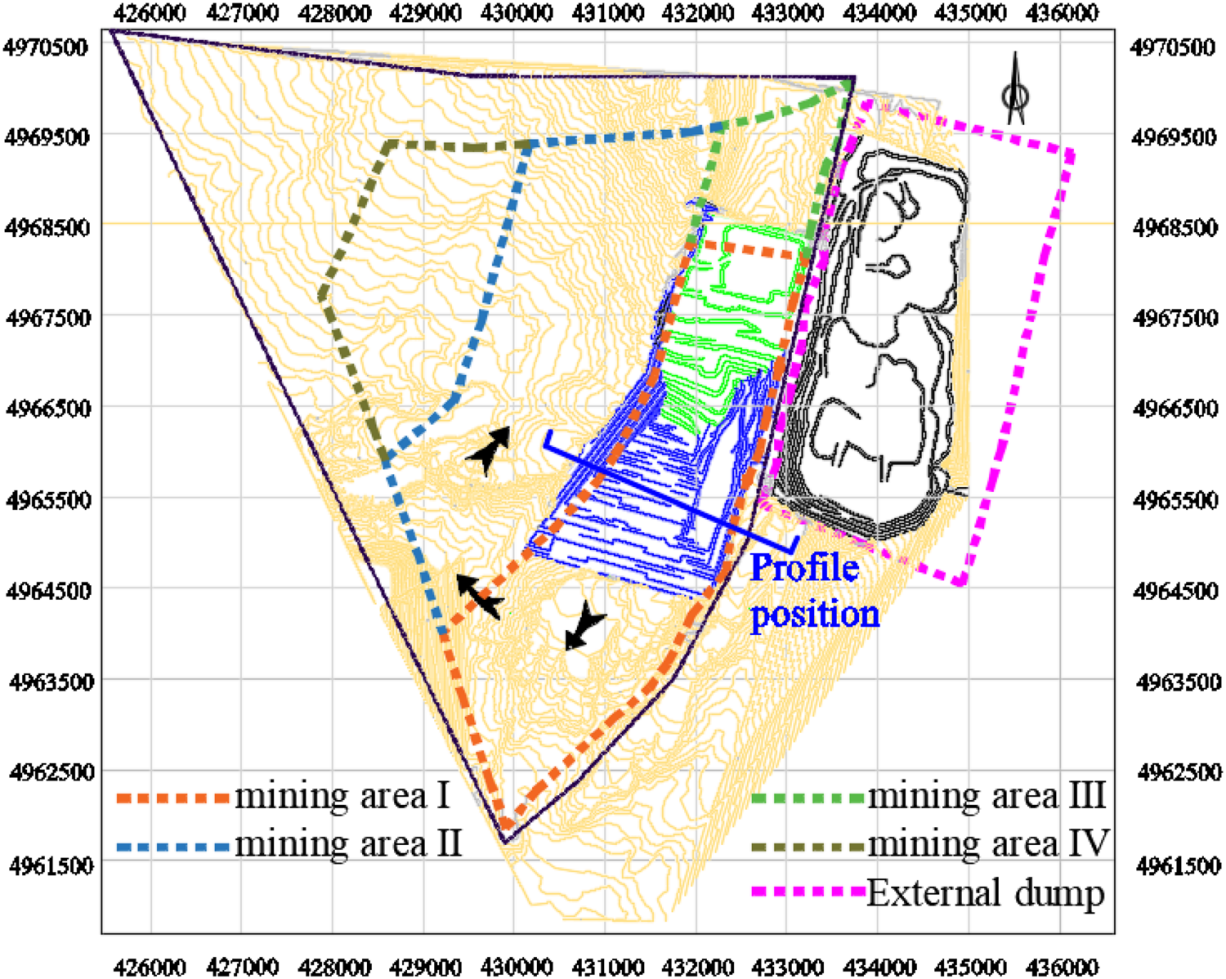
Division and mining sequence diagram of an open pit mine.

The selected open-pit coal mine has a large production capacity, and the raw coal produced is typically sold directly in the market, thus making the economic performance of the enterprise closely related to changes in coal prices. The main coal seam mined in this open pit mine is a single large thick inclined coal seam (Group B coal seam) with an average mining thickness of 69.43 m. According to the boundary elements of the open pit mine, the buried conditions of the coal seam and the size of the stripping ratio, combined with the characteristics of the mining technology adopted, the mining sequence is designed to be the first mining area → the third mining area → the fourth mining area, with the second mining area as the standby mining area. The division of mining areas and the mining sequence diagram are shown in Fig. 17.

**Figure 18:**
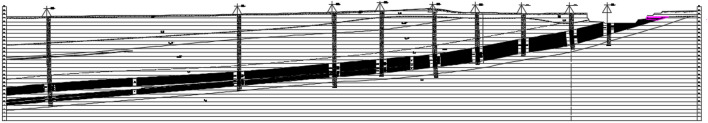
Cross section of an open pit mine.

The main coal seam of the open pit mine is a single inclined coal seam, and the shallow part is steep, deep and slow. The geological section is cut at the position shown in Fig. 17, and the profile is shown in Fig. 18.

The coal-rock mass in the study area is described using a block model to prevent memory overflow in the computer. The primary block size is set to 50*50*5 m, while the secondary block size is set to 25*25*1 m. The surface model and the mining boundary of the initial mining area are used to constrain the block model. The initial mining area contains a total of 697,985 blocks, which is sufficient for coal price forecasting within the desired period. From an economic perspective, the production capacity of the open-pit mine is regulated by coal prices. For the convenience of narration, let us suppose that the annual advance of the open pit mine is a constant 300 m/a. That is, the only way to expand the production capacity of an open pit mine is to increase the length of the working line. Of course, for an actual production process, the slope angle, thrust, working line length and other parameters are variable. This paper is simplified only for the purpose of theoretical research. Although it will not have a great impact on the research results, it still needs to be supplemented and improved by further research.

### Coal price forecast period dynamic adjustment effect test

As mentioned above, different boundary adjustment thresholds can be adopted according to the construction and development period of the open pit mine, that is, the value of α is a dynamic process. The coal price fluctuation trend in Fig. 11a is taken as the basis for the boundary adjustment, and the boundary adjustment period is set as 10 years. Due to practical engineering restrictions, it is assumed that the range of the length of the working line changing with the coal price does not exceed 20% of the current length of the working line. The comparison between the actual coal price and the preset coal price in each year of the adjustment period is shown in Table 3.

**Table 3:** Comparison of actual and preset coal prices during the adjustment period.

Year	Actual coal price (RMB)	The price actually used after adjusting boundary (RMB)	The range of coal price change	Boundary adjustment range	*α* value	Demand for boundary adjustment
2009	–	285.85	–	–	–	–
2010	340.93	340.93	16.16%	16.16%	− 0.24	Expansion
2011	408.23	408.23	16.48%	16.48%	− 0.25	Expansion
2012	332.99	332.99	− 18.43%	− 18.43%	0.29	Reduce
2013	237.07	266.39	− 28.80%	− 20.00%	0.52	Reduce
2014	172.07	213.11	− 35.40%	− 20.00%	0.67	Reduce
2015	110.27	170.49	− 48.26%	− 20.00%	0.96	Reduce
2016	190.03	190.03	11.46%	11.46%	− 0.14	Expansion
2017	353.40	228.04	85.97%	20.00%	− 1.00	Expansion
2018	347.09	273.64	52.21%	20.00%	− 1.00	Expansion
2019	313.28	313.28	14.48%	14.48%	− 0.20	Expansion
2020	286.01	286.01	− 8.70%	− 8.70%	0.07	Reduce

**Figure 19:**
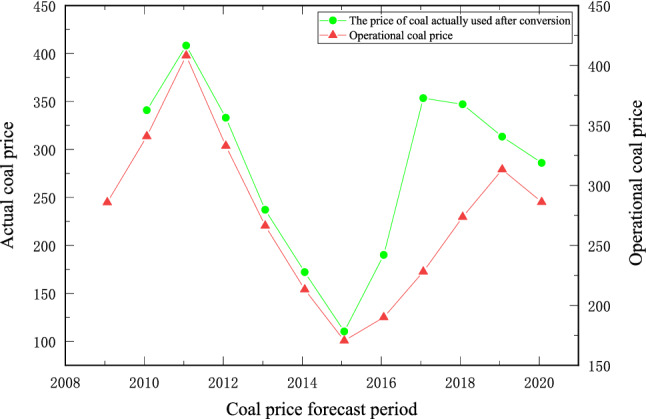
Comparison between the used and actual coal prices in the multistep boundary optimization design process.

As seen from Table 3, when the degree of achievement cannot meet the demand at a certain period during the adjustment period, it is necessary to adjust the open pit mine limit in time to meet the risk minimization demand. Figure 19 shows the comparison between the stage coal price and actual raw coal price in the process of the multistep boundary optimization method. The coal price used in each stage fits the trend of the actual coal price well. Except for the period of abnormal change in coal price, the coal price used in most stages is within the range of ± 20% of the actual coal price.

**Figure 20:**
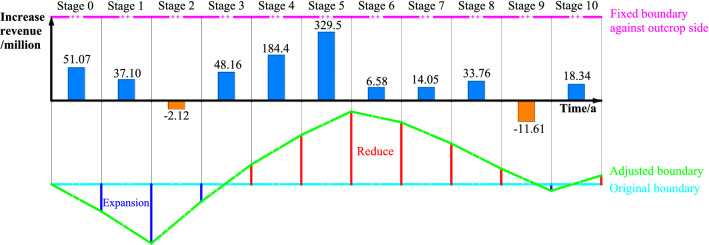
The change trend of boundary and profit value during the coal price forecast period.

The actual used coal price after conversion in Fig. 18 is applied to the boundary adjustment of the inclined coal seam open pit mine. Since the annual advance is assumed to be 300 m/a, the fluctuation of coal price can be dealt with through the change of the boundary. The production cost of the open pit mine is shown in Table 2. We easily obtain the following data: the cost of mining coal and postprocessing is approximately 20 yuan/m^3^, the cost of mining external discharge material is approximately 18 yuan/m^3^, and the cost of mining internal discharge material is approximately 14 yuan/m^3^. At the same time, according to the actual production data, raw coal profit is approximately 20% of the pit price. The boundary change and annual income increase during the boundary adjustment period are shown in Fig. 20.

Using the dynamic boundary adjustment method to make the boundary change closely follow the trend of the raw coal price effectively improves the economic benefits of enterprises. We calculate that in the whole 11-year coal price forecast period, using the method proposed in the paper, the cumulative increase in net profit of open pit mines is 709.23 million yuan. At the same time, we find that the method in this paper has strong applicability under the condition of large changes in coal price. However, when the coal price time series reaches a turning point, there may be a situation that leads to losses for enterprises. Further work is needed to determine whether and by how much the boundary should be adjusted when the inflection point occurs. However, in general, this method has good applicability to improving enterprise income and has certain theoretical value and practical significance.

## Discussion

In this paper, starting from the optimization design of coal limits under the fluctuation of coal prices, the ISSA–LSSVR method is applied to coal price time series prediction, and on the basis of the forecast of the next year's coal price, the adjustment range of the limit is calculated in reverse. Under the condition of controllable risks, the change trend of the limit is close to the coal price, which effectively improves the benefits of enterprises.

In terms of coal price time series prediction, this paper uses the sparrow search algorithm based on the intelligent group decision algorithm to determine the key parameters of a support vector machine prediction model and tries to determine the trend of coal price change with the help of this model. Considering that the traditional sparrow algorithm easily falls into local optima and the calculation complexity of SVR prediction is high, the ISSA algorithm based on tent chaos perturbation is used to analyse the parameters of the least squares support vector machine model, and the ISSA–LSSVR coal price time series prediction model is proposed. The forecast method is applied to the coal price series collected from 2010 to 2020. The results show that compared with the more traditional ARIMA prediction model, the proposed model can better match the change trend of coal prices and has higher prediction accuracy. However, when the coal price time series has abnormal fluctuations, low accuracy consistent with the existing prediction model will occur, so further research is needed to improve the prediction effect of the model on abnormal changes in value.

In the aspect of risk measurement of open pit limit design, entropy risk measurement is introduced into the effect evaluation of limit optimization. The positive and negative values of α are used to reflect the different risks faced by open pit mines. When α < 0, enterprises tend to take risks, and it is necessary to expand the limit of open pit mines to reduce the loss of profits. When α > 0, it tends to avoid risks, and at this time, it is necessary to reduce the open pit limit and reduce the loss profit of enterprises.

In the aspect of boundary optimization and adjustment, the coal price forecast results are applied to the boundary optimization and adjustment so that the boundary shows roughly the same trend as the coal price to improve the efficiency of enterprises. Taking a small open pit mine with an inclined coal seam as an example, the principle and method of limit adjustment are explained. At the same time, the method is applied to an open pit mine with an inclined coal seam, and the coal price trend in the forecast period is applied to boundary optimization adjustment, which brings a total income of 709.23 million yuan for the enterprise during this period. However, the method in this paper is not effective for adjusting the position of the price inflection point, and how to improve the return in this case needs further research.

In conclusion, the application of the ISSA–LSSVR coal price time series prediction method to open pit mine limit adjustment proposed in this paper has certain theoretical significance, but the prediction accuracy of the model and the effect of dealing with abnormal fluctuations still need to be improved. Considering the small number of time series samples used, the validity of the model under large-scale samples needs to be further tested. At the same time, the boundary adjustment method is not effective in predicting the inflection point, so further research is needed to put forwards a unified quantitative standard to realize the complete construction of the adjustment method. The next research work will focus on time series outlier processing, boundary adjustment scheme implementation system standardization and construction, and prediction model accuracy tests under large-scale samples.

## Conclusion

In this article, by introducing the flexible idea into the coal price prediction process, LSSVR prediction model is proposed to predict the coal price time series. Considering the given regularization term *C* and kernel function parameter *g* of traditional LSSVR, the prediction process is too subjective. The ISSA algorithm was used to optimize the above two parameters in the ISSA–LSSVR model. By taking the two parameters as the optimal position of the sparrow population, the optimal *C* and *g* values were obtained as 1.95 and 1.00 respectively. The accuracy test of the model shows that the calculation accuracy and error of the proposed algorithm are improved compared with the traditional ARIMA method, and the predicted results have a high degree of fitting with the actual values. Combined with the risk nesting property in the process of open-pit optimization design, the mathematical formula of boundary optimization problem is given in terms of cost minimization. By combining the coal price prediction results with the boundary optimization problem, the final mining boundary of the open-pit mine is dynamically adjusted according to the coal price fluctuation trend, and the risk of the preset profit index deviating from the actual scene is effectively dealt with. Taking an idealized small open-pit coal mine as an example, different risk aversion degrees are given according to different coal price fluctuations, and then the open-pit mine limits are adjusted. At the same time, taking an open pit mine in Xinjiang, China as an example, the coal price variation trend in the forecast period is applied to the boundary optimization adjustment. During the whole period, the adjustment of the state in most periods can bring considerable benefits to the enterprise. However, the method in this paper is not effective in adjusting the position of price inflection point, so it needs to be further studied to improve the return under such circumstances.

## Data Availability

The data used to support the findings of this study are available from the corresponding author upon request.
